# The relationship between observational scale and explained variance in benthic communities

**DOI:** 10.1371/journal.pone.0189313

**Published:** 2018-01-11

**Authors:** Alison M. Flanagan, Roger D. Flood, Michael G. Frisk, Corey D. Garza, Glenn R. Lopez, Nicole P. Maher, Robert M. Cerrato

**Affiliations:** 1 School of Marine and Atmospheric Sciences, Stony Brook University, Stony Brook, New York, United States of America; 2 School of Natural Sciences, California State University, Monterey Bay, Seaside, California, United States of America; 3 The Nature Conservancy, Long Island Chapter, Cold Spring Harbor, New York, United States of America; University of Sydney, AUSTRALIA

## Abstract

This study addresses the impact of spatial scale on explaining variance in benthic communities. In particular, the analysis estimated the fraction of community variation that occurred at a spatial scale smaller than the sampling interval (i.e., the geographic distance between samples). This estimate is important because it sets a limit on the amount of community variation that can be explained based on the spatial configuration of a study area and sampling design. Six benthic data sets were examined that consisted of faunal abundances, common environmental variables (water depth, grain size, and surficial percent cover), and sonar backscatter treated as a habitat proxy (categorical acoustic provinces). Redundancy analysis was coupled with spatial variograms generated by multiscale ordination to quantify the explained and residual variance at different spatial scales and within and between acoustic provinces. The amount of community variation below the sampling interval of the surveys (< 100 m) was estimated to be 36–59% of the total. Once adjusted for this small-scale variation, > 71% of the remaining variance was explained by the environmental and province variables. Furthermore, these variables effectively explained the spatial structure present in the infaunal community. Overall, no scale problems remained to compromise inferences, and unexplained infaunal community variation had no apparent spatial structure within the observational scale of the surveys (> 100 m), although small-scale gradients (< 100 m) below the observational scale may be present.

## Introduction

Understanding the role of scale in freshwater, marine, and terrestrial communities is vital in ecology [1–3]. Scale in processes, data collection, and analysis [4] has relevance to virtually all ecological investigations with its ability to influence the outcome or conclusion of a study [1], [5]. Central to the ecological significance of scale is hierarchy theory [6], which has been used to conceptualize the processes by which multiple, scale-dependent, and potentially interacting phenomena contribute to the biological heterogeneity that is ultimately observed in nature [7], [8].

In benthic environments, geomorphology tends to be complex resulting in conceivably abrupt changes in faunal communities, a pattern expected from hierarchy theory [9]. Careful examination of biological structuring through the lens of hierarchy theory may help to deepen our understanding of the ecological significance of scale. For example, a common practice for ecologists is to simplify nature [2] using habitat classification [8,10–13] as an approach for managing ecosystems. Most habitat classification approaches are based on hierarchy theory in that they are designed to characterize habitat features from large (e.g., basins, boulder fields, etc.) to small (e.g., burrows, sand waves, etc.) spatial scales [8]. Thus, hierarchy theory comprises a natural link between scale, habitat classification, and ultimately spatial analysis.

The observational scale of an ecological study is defined by the grain or size of the sampling unit, the sampling interval (or lag in time series analysis) between samples, and the extent of the study [1,4,14]. Many investigators have focused on grain and extent, and the effects of varying these factors are well known in benthic systems [15,16]. Less understood is the effect of varying the sampling interval (i.e., the geographic distance between samples). The sampling interval is generally limited by the cost and effort of collecting and processing community data [17]. Sampling at small intervals may leave out important controlling environmental factors while sampling at large intervals may make it difficult to identify patterns in the fauna [1]. A relevant question to ask is how much does the sampling interval affect biotic-environmental inferences, since once a sampling regime is chosen, spatial variation occurring at smaller intervals cannot be investigated. Sampling and analysis scale have been historically somewhat arbitrary [18] and potentially biased through anthropocentric perceptions of nature–a problem aptly referred to as “scale arbitrariness” by Wiens (1989) [1]. So, the scales being used in scientific studies may not reflect the scales that are important and/or relevant to the animals and communities that are being investigated.

Field-based sampling methods for examining benthic communities have traditionally involved *in situ* sampling with grab samplers, corers, and dredges. The distance interval between these samples defines the smallest scale over which the data set can provide information about biotic-environmental relationships. Below that distance, small-scale variations are present [14]. These are presumably due biotic interactions [7], especially at scales approaching the organisms themselves [19], along with small-scale variability in measured environmental variables, other unmeasured environmental factors that become important at small scales, and measurement error.

Recently, uncovering spatial patterns in benthic communities through the application of geospatial tools (e.g., sonar, high-resolution videography, etc.) has emerged as a field in its own right [20–22]. The key advantage to geospatial methods, especially sonar, is that they can provide near to full, high-resolution coverage of the areas surveyed versus the seafloor “snapshots” obtained by traditional *in situ* sampling [22]. In addition, with the proliferation of techniques for the analysis of sonar data (e.g., acoustic segmentation via visual, supervised, and/or unsupervised classification), acoustically-defined habitats (referred to as “provinces” in the present study) or areas of the seafloor consisting of apparently homogeneous geophysical conditions can be readily derived and used in analysis. Despite its clear potential, not all acoustic features that appear to characterize distinct regions are relevant to benthic community structure [23–26]. For instance, regions defined using acoustic data in areas consisting of unconsolidated sand and gravel sediments did not correspond well to infaunal assemblage boundaries [24]. Further, significant but weak associations between sonar backscatter and community structure have also been observed [25]. Beyond the “seascape” scale, other scales at which sonar data should be analyzed to provide useful information on community structure are unclear. Yet, scale selection for the purposes of analysis can be as critical as the observational scale is for identifying relationships [4].

Multiscale ordination (MSO) extends spatial statistics to examining the structure of biotic-environmental relationships by inserting multivariate regression results from a direct gradient method, such as redundancy analysis (RDA), into an empirical variogram [27–29]. MSO is distance-based, where the spatial structure in a data set is scrutinized using an empirical variogram, a plot of the sum of the mean squared species-specific differences between pairs of samples against their geographic distance [7,27]. MSO takes the fitted and residual output of a nonspatial regression analysis and partitions it at different spatial scales or distance intervals in geographic space. Results are then examined critically for spatial patterns. When fitted and residual variograms are combined and compared to the empirical variogram, they can indicate whether the regression model is misspecified or whether the biotic-environmental relationship changes with spatial scale. The presence of a small-scale trend in the residual variogram may suggest the presence of potentially important species interactions, while a trend at a large spatial scale may be evidence that one or more environmental variables have been missed. Furthermore, spatial correlation structure in the residuals creates non-independence problems that can undermine the data analysis [14,30].

The goal of this study was to use multiscale ordination to examine the spatial structure and biotic-environmental relationships of infaunal communities using six data sets ranging from freshwater to near marine conditions. It was envisioned that identifying characteristics common to all six data sets would provide valuable and perhaps general insights. Analysis of the data focused on quantifying the fraction of community variation that is below the sampling interval, and therefore below the resolution of the study, estimating the amount of community variation explained by commonly used *in situ* and seascape-scale explanatory variables, determining whether explanatory variables identified during the model selection process removed spatial structure in the data, or whether unresolved and therefore problematic structure remained, and assessing the effectiveness and limitations of the sampling and analytical scales used. We hypothesized that small scale community variability would be large and impose a limit on the amount of variation that could be explained by *in situ* and seascape-scale environmental variables. We predicted that spatial structure within the observational scale of the studies would be present but effectively explained by a few common environmental variables. Additionally, it was expected that incorporating spatial scale elements would alter the perceived effectiveness of how well these environmental variables explained community variation, and the altered perception would be large enough to potentially affect research and management directions.

## Materials and methods

**Fig 1:**
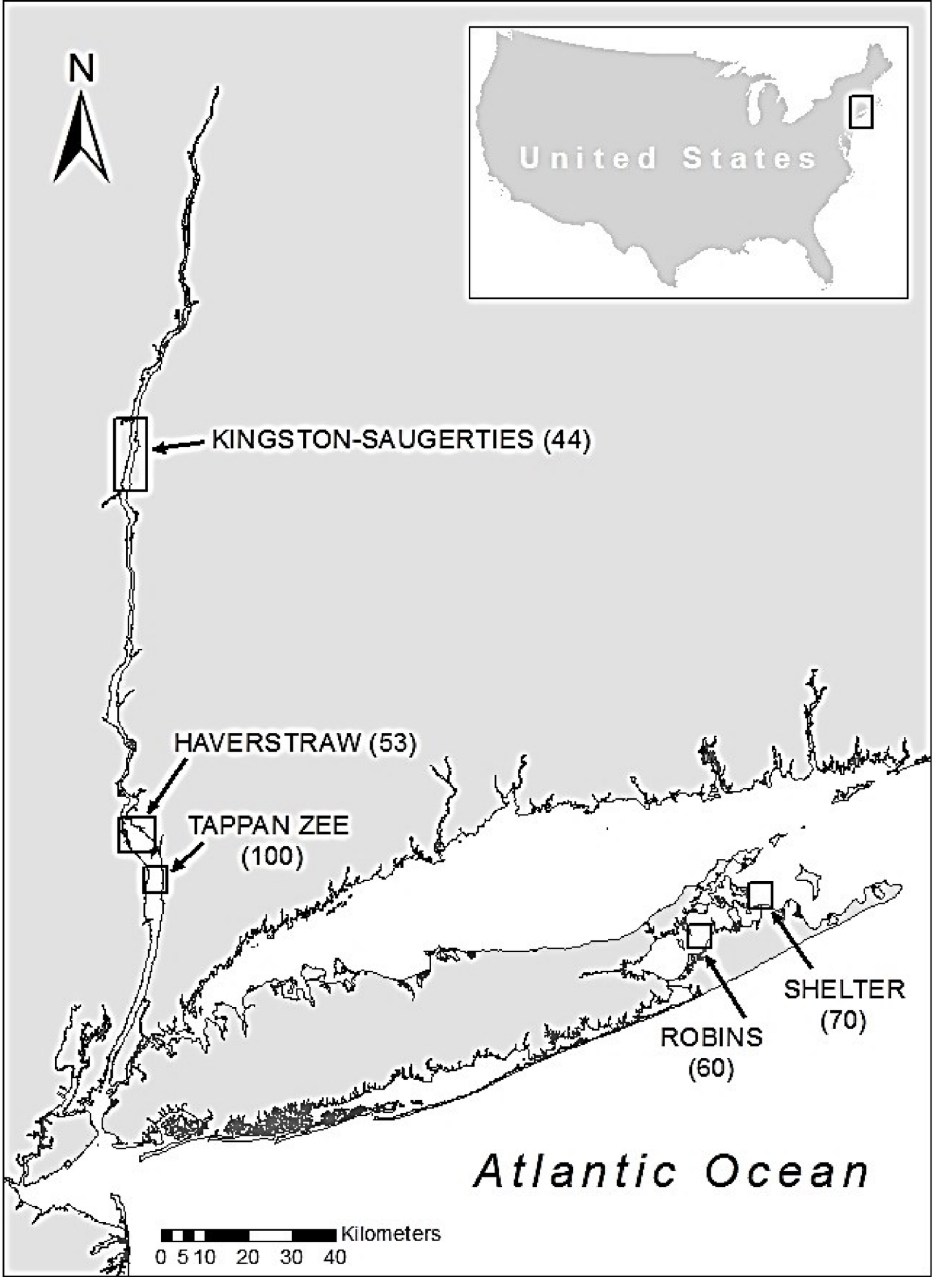
Map of study areas including the number of samples within each location in parentheses.

This study analyzed six benthic biotic-environmental data sets from five study locations in New York, USA including three locations within the Hudson River Estuary and two in eastern Long Island’s Peconic Estuary (Fig 1 & Table 1). The five locations represented a range of benthic environments from freshwater (Kingston-Saugerties) to brackish (Haverstraw Bay and Tappan Zee) to near marine (Robins Island and Shelter Island). Sites are moderate in area, ranging from 3.3 to 16.5 km^2^.

**Table 1:** Data inventory table with references. RPD and LOI refer to the apparent redox potential discontinuity depth and sediment organic matter measured by loss on ignition, respectively.

Study Location (Sample Size)	Sampling Device	Water Depth	RPD	LOI	Grain Size Reference	Surficial Percent Cover	Acoustic Province Reference
Kingston-Saugerties '01 (44)	Petite PONAR grab	✔	-------	✔	[41]	-------	[36]
Kingston-Saugerties '02 (44)	Petite PONAR grab	✔	-------	✔	[41]	-------	[36]
Haverstraw (53)	Modified van Veen grab	✔	✔	-------	[40]	-------	[38]
Tappan Zee (100)	Modified van Veen grab	✔	-------	-------	[40]	✔	[36]
Robins (60)	Modified van Veen grab	✔	-------	-------	[40]	✔	[39]
Shelter (70)	Modified van Veen grab	✔	-------	-------	[40]	✔	[39]

### Acoustic provinces

**Fig 2:**
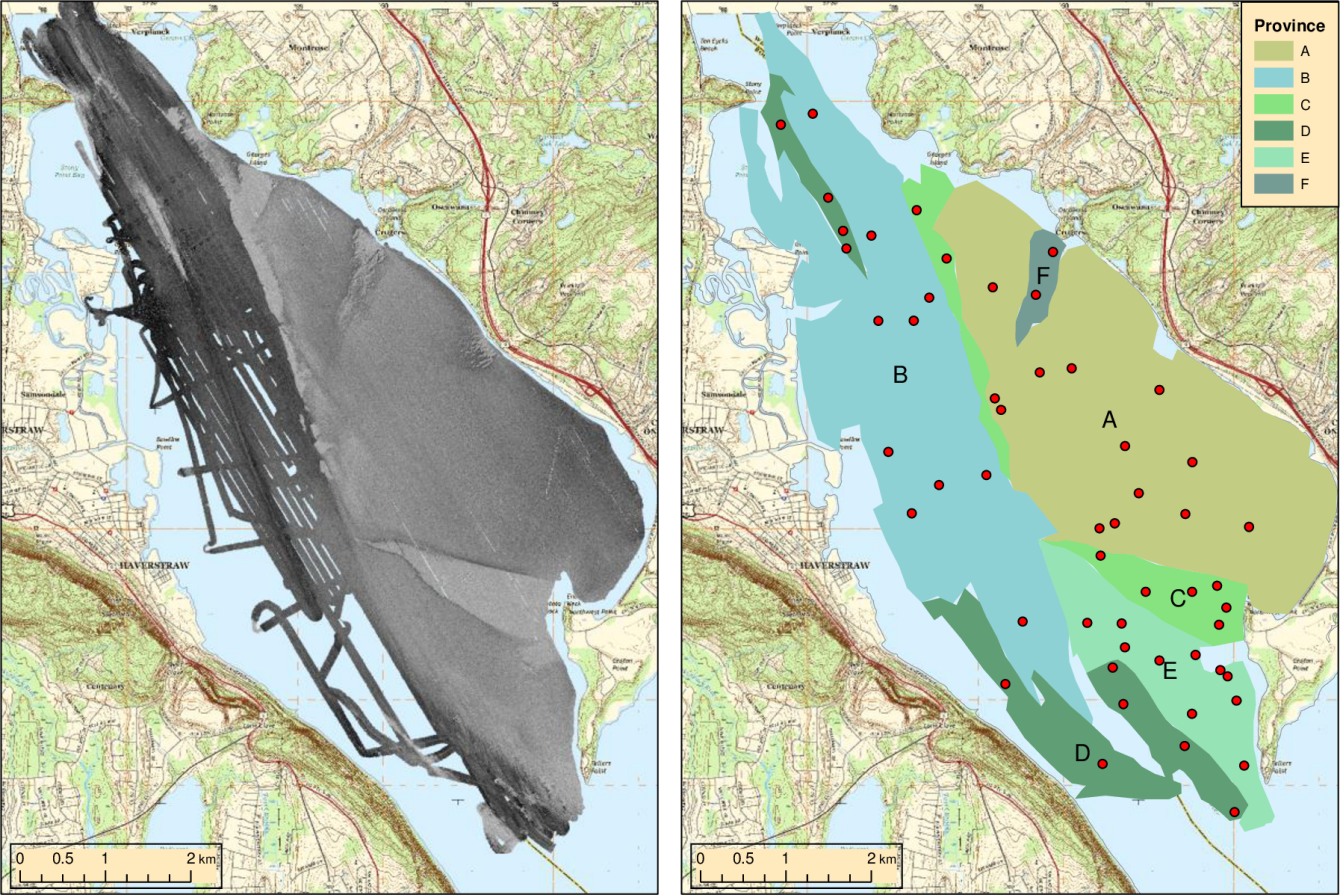
Example of categorical acoustic provinces (right) created from backscatter data (left) from the Haverstraw Bay study site. Points indicate the location of sampling stations. Basemap from https://nationalmap.gov.

Seascape or habitat-scale environmental data were generated by segmenting each site into regions of the seafloor with apparently homogeneous geophysical conditions (Table 1). This was based primarily on visual interpretation of backscatter intensity and texture in multibeam and/or sidescan sonar surveys (e.g., Fig 2), assuming that the sonar data represented proxies for a variety of natural features and phenomena (e.g., exposure to subtidal currents, vulnerability to sedimentation, compaction, shell hash) that could govern faunal patterns in the benthos [22,23,31–35]. These regions will be referred to as acoustic provinces in this study. Backscatter data were obtained for the Haverstraw Bay and Robins Island sites with a Kongsberg Simrad EM 3000D multibeam sonar system (300 kHz). Multibeam data were gridded on a 1m resolution with horizontal accuracy of 1m. Sidescan sonar data were acquired for the Kingston-Saugerties, Tappan Zee, and Shelter Island areas using an Edgetech DF-1000 or 272 DT sidescan sonar system (100 kHz), gridded on a 2 m resolution. Chirp sub-bottom seismics, sediment cores, and grain size data from grabs were also used as supplementary information for the identification of acoustic provinces at Kingston-Saugerties and Tappan Zee by Bell et al. (2000) [36], and their province maps for these sites were adopted. Province identification was carried out prior to and independently of any faunal sampling or analysis, and no backscatter or supplementary data used in the identification of provinces were used in the subsequent faunal analyses. Visual interpretation resulted in 10 provinces for Kingston-Saugerties [36,37] 5 provinces for Haverstraw Bay [38], 10 for Tappan Zee [36], and 6 and 7 provinces, respectively, for the Robins Island and Shelter Island sites [39]. Provinces were represented as categorical variables in subsequent data analyses. Although character designators (A-J) were used to identify provinces within a site (e.g., Fig 2), habitat types did not map across sites thereby making general between-site comparisons impossible. For example, there were no freshwater channel deposits at any other site than Kingston-Saugerties. In addition, sampling years and seasons also differed making any potential between-site analysis confounded by time.

### In situ community and environmental data

Faunal and sediment samples were collected *in situ* using a modified Van Veen grab (20 × 20 cm) at Haverstraw Bay (n = 51), Tappan Zee (n = 100), Robins Island (n = 60), and Shelter Island (n = 70) and a petite PONAR grab (15 × 15 cm) at Kingston-Saugerties (n = 44; 3 pooled samples per station). Sampling locations were random, but stratified by province, to ensure full coverage of the habitats present. The Kingston-Saugerties site was sampled in Fall 2001 and Spring 2002 (designated with a suffix ‘01 and ‘02 in results), and each data set was analyzed separately. Water depth was recorded at the time of sample collection. A sediment subsample for grain size analysis was drawn from each grab sample and the remainder washed through a 0.5 mm sieve for fauna. Macrofauna remaining on the sieve were preserved in 10% buffered formalin. In the lab, grain size analysis to estimate percent gravel, sand, and mud was conducted using the technique described by Folk (1974) [40] for all locations except Kingston-Saugerties, where the hydrometer method for particle size analysis was used instead [37,41]. Faunal samples were sorted under a dissecting microscope, identified to species whenever possible, and species abundance per sample was enumerated.

Surficial percent cover data were obtained from analysis of images extracted from seabed surface videos recorded at Tappan Zee (n = 100), Robins Island (n = 60), and Shelter Island (n = 70) using a 2 megapixel Seatrex HD underwater camera mounted on an aluminum tripod. Videos captured 17.5 × 30 cm areas of the seafloor except at Tappan Zee where the camera had to be lowered closer to the bottom since visibility was limited due to the extremely turbid conditions at this location. Recordings from Tappan Zee covered 13.5 × 23.5 cm areas of the seafloor. Percent cover estimates of the visible surficial components present in each image (e.g., shell, pebble, fauna, etc.) were obtained by supervised maximum likelihood classification analysis in ArcGIS 10.1 (ESRI, Redlands, CA).

### Empirical variograms

Spatial variability in the species assemblages was examined by constructing an empirical variogram of the multivariate faunal data for each site [27]:

$$\gamma (h)=\sum_{i=1}^{s}\frac{1}{2n_{h}}\sum_{a,b|h_{ab}\approx h}{\left(x_{ia}-x_{ib}\right)}^{2}$$ (1)

where *γ*(*h*) is the empirical variance in the faunal data at distance class *h*, *x*_*ia*_ and *x*_*ib*_ are Hellinger transformed abundances for species *i* (*i* = 1 *to s*) in samples *a* and *b*, respectively, and the inner summation is over all pairs of samples separated by a geographic distance of approximately *h*. The Hellinger transformation is the square root of the relative abundance of each species in a sample [42]. As such, it focuses the analysis on compositional differences and downplays the influence of highly abundant species to prevent them from dominating the analysis. In addition, when used in conjunction with Euclidian distance, the ecological distance measure utilized here and in the multivariate regression analyses presented below, it produces good representations of ecological dissimilarity [42]. Summing Eq 1 for all pairs of samples, instead of a distance class subset, yields *s*^2^ the total sample variance [43].

A plot of *γ*(*h*) vs. distance classes *h* is called a variogram plot and shows how variance between samples changes with spatial scale. Changes in *γ*(*h*) with distance indicate the presence of spatial patterns in community structure. Often when spatial structure is present, *γ*(*h*) increases from some minimal value at small distances, indicating that community differences increase as samples are collected further apart. This is not the only pattern possible, and variograms with no trend, negative trends, periodic trends, and more complex patterns have been observed [14,44–46],. Important features of the multivariate variogram relevant to the present study include the *sill*, which is the value of the variance where the variogram plot levels out (if it exists) and the *nugget* or *nugget effect*, the value of *γ*(*h*) at *h* = 0 [14,27]. If a sill is present, the *range* indicates the distance at which community variance is no longer increasing. For reference, definitions of the variogram terms and their ecological interpretations are provided in Table 2. The quantity *γ*(*h*) is termed the semivariance in most references on spatial statistics, although Bachmaier and Backes (2008) [43] suggest that the prefix “semi” is applied incorrectly. Because of its common usage, it will be called semivariance in the present study. Variograms were created using the rda(), mso(), and msoplot() functions in the vegan package of R (R Foundation for Statistical Computing, Vienna, Australia). The code for mso() and msoplot() was created and first published by Wagner (2004) [28].

**Table 2:** Variogram nomenclature [14,47,48]. Symbols refer to model parameters in Eqs 2–6.

Term	Symbol	Variogram Definition	Ecological Interpretation
Nugget	*c*_*0*_	The y-intercept of a variogram at distance *h* = 0.	Variance that occurs at a spatial scale smaller than the sampling interval. Estimates the community variance that is below the resolution of a survey.
Sill	*c*_*0*_ *+ c*_*1*_	The value of the variance where the variogram levels off.	Community variance of pairs of samples that are separated at large enough geographic distances that they are spatially independent.
Range	*a* for Eqs 4 & 5	The geographic distance where the sill is reached.	The geographic distance beyond which community structure in pairs of samples are spatially independent. Spatial structure is present at distances below the range.
	*h* such that		
	*ɣ*(*h*) = 0.95(*c*_*0*_ *+ c*_*1*_) for Eqs 2, 3, & 6		
Maximum Extent	*h*_*max*_	The largest geographic distance between two samples in a study.	Identical to the variogram definition; this simply refers to the maximum geographic distance between two samples in a study.

### Estimating small-scale variation

Empirical variograms created for each site were fit to a number of common models utilized in spatial statistics [48]. Since no *a priori* guidance or mechanistic reason was found to choose one model over the others, multiple models with a variety of characteristics, including with/without an inflection at small spatial scales and with/without an asymptotic sill, were fit. The models included the exponential, Gaussian, spherical, piecewise linear, and logistic as follows:

$$\gamma (h)=c_{0}+c_{1}\left(1-e^{-\frac{h}{a}}\right)$$ (2)

$$\gamma (h)=c_{0}+c_{1}\left(1-e^{-\frac{h^{2}}{a^{2}}}\right)$$ (3)

$$\gamma (h)=\left\{ \begin{array}{c} c_{0}+c_{1}(\frac{3}{2}\frac{h}{a}-\frac{1}{2}\frac{h^{3}}{a^{3}})\;for\;0<h\leq a\\ c_{0}+c_{1}\;for\;h\geq a \end{array}\right.$$ (4)

$$\gamma (h)=\left\{ \begin{array}{c} c_{0}+\frac{c_{1}}{a}h\;for\;0\;<h\leq a\\ c_{0}+c_{1}\;for\;h>a \end{array}\right.$$ (5)

$$\gamma (h)=c_{0}+\frac{c_{1}ah^{2}}{1+ah^{2}}$$ (6)

In the above equations, *c*_0_ is the nugget effect, *c*_0_ + *c*_1_ is the sill, and *a* defines the rate and, in the case of the spherical and piecewise linear models, the range at which the sill is reached. For asymptotic models (exponential, Gaussian, and logistic), the range is estimated as the value of *h* where *γ*(*h*) equals 95% of its sill [48].

Models were fit by weighted least squares, as suggested by Cressie (1993) [47], with weighting factors defined by the number of sample pairs in each distance class. Only distance classes less than half the maximum extent of the site (i.e., *h*_*max*_*/2)* were utilized, since beyond that distance, sampling locations in the center of the site can no longer contribute to variance estimates, leading to potential bias [27,47]. In the present application, distance classes were set to intervals of 0.25, 0.50, or 0.75 km. The interval was selected for each data set to have plots with about 10 *γ*(*h*) values. This usually allowed the distance classes to contain >30 sample pairs, a recommendation suggested by Journal and Huijbregts (1978) [49]. Weighted least squares was carried out using function nls() in the stats package of R (R Foundation for Statistical Computing, Vienna, Australia). It was assumed that the residuals were approximately normally distributed, and model selection was achieved using the small sample, bias-adjusted version of Akaike’s Information Criterion (AICc) [50,51].

Distributional properties of parameter estimates in variogram fits are “not well understood” [47]. Since individual sample points are reused multiple times in creating the variogram (Eq 1), independence assumptions are violated, and application of procedures such as bootstrap, jackknife, or cross-validation to determine errors in parameter estimates are not valid [47]. As a result, no error analysis of parameter estimates was possible.

### Multiscale ordination

Multiscale ordination (MSO) extends spatial statistics to examining the spatial structure of biotic-environmental relationships by inserting regression results into the variogram [27–29]. It does this by partitioning the *x*_*ia*_ and *x*_*ib*_ pairs in Eq 1 into fitted and residual parts ${(\hat{x}}_{iafit}+{\hat{x}}_{iares})$ and ${(\hat{x}}_{ibfit}+{\hat{x}}_{ibres})$, respectively. Substituting these into Eq 1 leads to:

$$\gamma (h)=\overset{s}{\underset{i=1}{\sum }}\frac{1}{2n_{h}}\underset{a,b|h_{ab}\approx h}{\sum }\left[{\left({\hat{x}}_{iafit}-{\hat{x}}_{ibfit}\right)}^{2}+{\left({\hat{x}}_{iares}-{\hat{x}}_{ibres}\right)}^{2}+2({\hat{x}}_{iafit}-{\hat{x}}_{ibfit})({\hat{x}}_{iares}-{\hat{x}}_{ibres})\right]$$

or

$$\gamma (h)=\gamma_{fit}(h)+\gamma_{res}(h)+\gamma_{cross}(h)$$ (7)

The first two terms on the right-hand side are variograms of the fitted and residual values. The third term is twice the covariance between the fitted and residual differences for distance class *h* [27]. Multivariate regression estimates of biotic-environmental relationships can be obtained by redundancy analysis (RDA) for small to moderate environmental gradients or canonical correspondence analysis (CCA) for large gradients [28]. Detailed steps to implement this procedure are available in Borcard et al. (2011) [52] and Legendre and Legendre (2012) [14].

In the present study, estimates of predicted and residual Hellinger transformed abundance values were obtained by RDA. RDA is a multivariate method that combines multiple linear regression with ordination. A parsimonious set of explanatory environmental variables was identified by sequentially adding variables in a forward selection process [53]. Candidate variables for each site included water depth, grab penetration depth, apparent redox potential discontinuity (RPD) depth, grain size (% gravel, sand, and mud), sediment organic content measured by loss on ignition (LOI), surficial percent cover (shell, seaweed, and other materials observed in bottom images), and categorical variables as binary 1/0 values representing each acoustic province. At each step in the process, the environmental variable explaining the largest amount of faunal variability was selected, and its effect removed before the next best fitting variable was considered. Variables identified by forward selection were trimmed back to a smaller set by the AICc stopping criterion [54].

Categorical province variables were also combined into smaller sets (e.g., A&E combined, B&D combined, etc.) and regressions evaluated by the AICc criterion to ensure that the smallest number of distinct provinces was selected. Results from multivariate regression tree (MRT) analysis [55] were used to guide the formation of sets of provinces to avoid trying all 2^n^ - 1 unique combinations. Forward selection in RDA was carried out in Canoco 4.5 (Microcomputer Power, Ithaca, NY, USA), and variograms of regression results with the final set of explanatory variables were created using the rda(), mso(), and msoplot() functions in the vegan package of R (R Foundation for Statistical Computing, Vienna, Australia). MRT was run using the mvpart package in R (R Foundation for Statistical Computing, Vienna, Australia).

Following MSO, the spatial structure of the variogram components generated by RDA fitting was analyzed for scale dependence in the biotic-environmental relationship, stationarity of the residuals, and spatial autocorrelation in the residuals using methods in Wagner (2003) [27]. Scale dependence in the biotic-environmental relationship was tested by constructing a Bonferroni-corrected point confidence interval around *γ*(*h*) and determining if the sum of the variograms *γ*_*fit*_(*h*)+*γ*_*res*_(*h*) lies wholly within it. If any points in the sum lie outside then *γ*_*cross*_(*h*) is significantly different from 0 at specific distance classes, and the biotic-environmental relationship is scale-dependent. Stationarity of the residuals was examined by determining whether *γ*_*res*_(*h*) reached a sill after short distances and remained there over most of the range. Presence of a spatial trend in *γ*_*res*_(*h*) over large distance classes indicates that important environmental variables were missing in the RDA model. Spatial autocorrelation in the residuals was tested by a series of Bonferroni-adjusted Mantel tests [56] between the distance matrix formed by the residuals and a geographic distance matrix at each distance class interval. The Bonferroni correction set the significance level of the tests at *α*/*n* where *α* = 0.05 and *n* = the number of distance classes. A significant outcome for a distance class indicates that spatial correlation is present in the residuals [7,28]. The scale dependence and spatial autocorrelation analyses are built into the msoplot() function in the vegan package of R (R Foundation for Statistical Computing, Vienna, Australia).

### RDA performance at local vs. habitat-level scales

Variogram measures were recomputed to quantify the extent the RDA models explained local vs. habitat-level community variation. This analysis also assessed the ability of acoustic provinces to serve as proxies for habitats within the study areas. The variogram function mso() was modified slightly to replace the geographic distance matrix between pairs of samples with a matrix of two “distance” classes: within (1) and between (2) acoustic provinces. The output provided the variogram estimates for *γ*, *γ*_*fit*_, and *γ*_*res*_ for each “distance” class. Assuming that small-scale variation in the data cannot be explained by the RDA models’ biotic-environmental relationships, estimates of the nugget effect were subtracted from *γ*_*res*_ to assess model performance of only that part of the variation within the observational scale of the surveys.

## Results

### Summary of faunal and environmental data

An abundant and diverse fauna occurred at each study site. Mean abundance per m^2^, mean species density per sample, and species richness at each site are summarized in Table 3. Species richness varied from 25 taxa at Haverstraw Bay to 95 taxa at Shelter Island. The study sites represented a wide range of benthic environments ranging from freshwater at Kingston-Saugerties, to mesohaline at Haverstraw Bay and Tappan Zee (salinity ranges of 8.5–14.5 during sampling), to almost marine conditions at Robins Island and Shelter Island (salinity ranges of 28.5 to 31 during sampling). Average water depth varied from 5.7 to 9.9 m among study locations, and depth measurements varied widely at sampling stations within study areas (Table 4). The greatest variation in depth occurred at Kingston-Saugerties with sampling stations ranging from 1 to 22 m. The smallest variation was at Shelter Island with a range of 3 to 11 m. The sediments across all locations were mostly sandy or muddy and rarely contained substantial gravel, with some notable exceptions at individual sampling stations (Table 4). Nevertheless, the grain size range varied broadly; for example, percent mud ranged from less than 4% to greater than 83% within each study area.

**Table 3:** Summary of faunal data including mean abundance of all taxa per m^2^, mean species density per sample, and overall species richness at each study location.

Study Location	Number of samples	Mean abundance	Mean species density	Species richness
Kingston-Saugerties '01	44	5776	10	47
Kingston-Saugerties '02	44	3849	12	54
Haverstraw Bay	51	2643	9	25
Tappan Zee	100	2575	11	40
Robins Island	60	7063	25	71
Shelter Island	70	18534	24	95

**Table 4:** Summary of environmental data. Mean values (and range) of the grab-scale environmental variables acquired via *in situ* sampling. RPD and LOI refer to the apparent redox potential discontinuity depth and sediment organic matter measured by loss on ignition, respectively.

Study Location	Water Depth (m)	% Gravel	% Sand	% Mud (Silt-Clay)	RPD (cm)	LOI (%)
Kingston-Saugerties '01	9.9 (1.3–22.0)	-------	67.8 (11.6–99.8)	32.2 (0.2–88.5)	-------	2.9 (0.6–6.6)
Kingston-Saugerties '02	9.7 (1.1–22.0)	-------	66.3 (11.6–99.8)	33.1 (0.2–88.5)	-------	3.0 (0.6–6.6)
Haverstraw	6.4 (2.8–19.9)	14.2 (0–92.3)	19.39 (1.5–65.7)	66.0 (3.3–98.1)	1.0 (0–5.0)	-------
Tappan Zee	7.0 (2.7–12.2)	9.1 (0–64.4)	13.0 (1.1–60.8)	77.9 (3.4–98.9)	-------	-------
Robins	9.5 (3.6–18.3)	0.5 (0–8.5)	54.2 (11.4–98.6)	45.3 (1.3–88.4)	-------	-------
Shelter	5.7 (2.6–11.1)	14.2 (0–63.8)	77.0 (4.3–99.0)	8.8 (0.2–83.8)	-------	-------

The surficial percent cover classes from maximum likelihood analysis of seafloor images varied moderately among study locations (Table 5). A total of 5 cover classes were identified at Tappan Zee, with the majority of the sampling stations at this site composed of mud, silty shell, or other silt-covered material. Robins Island and Shelter Island had 9 and 14 cover classes, respectively. Surficial sediments at these two sites were predominantly combinations of rock, pebble, sand, mud, shell, shell hash, and silty material. Occasionally, biotic cover consisting of live slipper snails (*Crepidula fornicata*) and sponges (*Microciona porifera*) was present.

**Table 5:** Percent surficial cover from maximum likelihood analysis of images extracted from underwater video surveys at the Tappan Zee, Robins Island, and Shelter Island locations.

Percent Cover Class	Abbreviation	Study Location (number of classes)		
		Tappan Zee (5)	Robins Island (9)	Shelter Island (14)
		Mean % ± 1 SD	Mean % ± 1 SD	Mean % ± 1 SD
**Sand**	PCSa	-	-	40.19 ± 43.17
**Mud**	PCMu	97.60 ± 9.63	81.88 ± 37.11	3.17 ± 14.2
**Shell Fragment**	PCShFg	-	1.65 ± 3.12	1.58 ± 2.91
**Shell**	PCSh	0.08 ± 0.61	0.26 ± 1.17	7.10 ± 20.68
**Rock**	PCR	-	-	0.67 ± 2.85
**Pebble**	PCPb	-	-	1.19 ± 8.18
**Seaweed**	PCSw	-	0.44 ± 2.35	4.94 ± 8.74
**Silty Shell**	PCSiSh	0.97 ± 8.36	0.66 ± 2.41	4.85 ± 16.01
**Shell Pebble**	PCShPb	-	-	10.53 ± 25.53
**Muddy Sand**	PCMuSa	-	14.74 ± 33.51	13.5 ± 28.00
**Silty Material**	PCSiCovered	1.07 ± 3.61	-	-
**Anthropogenic**	Anthro	-	-	0.004 ± .040
**Unknown**	Unk	0.21 ± 1.16	0.01 ± 0.05	0.09 ± 0.67
**Microciona prolifera**	Mpor	-	0.02 ± 0.10	0.14 ± 0.32
**Crepidula spp.**	Crep	-	0.15 ± 0.84	11.87 ± 27.28

### Empirical variograms of community data and estimates of small-scale variation

**Fig 3:**
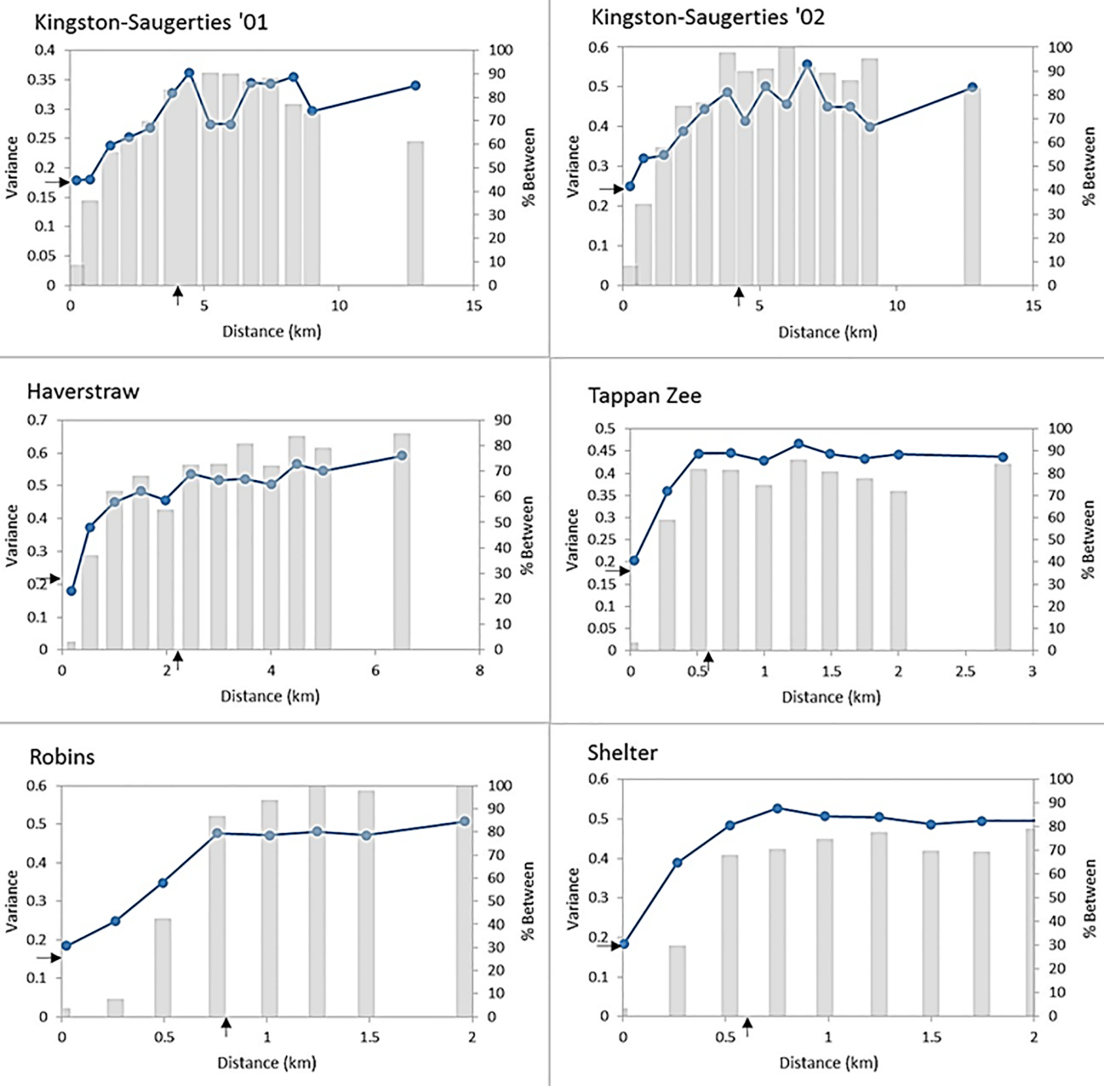
Empirical multivariate variogram of community data (points) and the percentage of sample pair comparisons between acoustic provinces (bars). Distance intervals ranged from 0.25 to 0.75 km among study locations. Arrows on the vertical and horizontal axes indicate nugget and range estimates, respectively.

The emprirical variance *γ*(*h*) increased with geographic distance class across all study locations, indicating that spatial structure was present in the species assemblages (Fig 3). At small distance classes, *γ*(*h*) was dominated by pairs of samples that were within the same acoustic province, and *γ*(*h*) reached a sill once it became dominated by between-province sample pairs at large distance classes. The shape of *γ*(*h*) was smooth for the Haverstraw Bay, Tappan Zee, Robins Island and Shelter Island sites, but more variable in the Kingston-Saugerties data sets. The pattern for Kingston-Saugeties probably reflects the elongated north-south structure of this study area in the Hudson River where short east-west distances can place sample pairs in different provinces but long north-south distances can still lead to within-province pairs contributing to *γ*(*h*).

Based on estimates of the nugget effect in the variogram models, small-scale variability represented a substantial fraction of the overall community variance across all data sets, ranging from 36 to 59% of the total faunal variance (Table 6). A piecewise linear model was selected for 2 of the 6 data sets (Kingston-Saugerties ‘01, Robins Island), a spherical model for 3 data sets (Kingston-Saugerties ‘02, Tappan Zee, and Shelter Island), and an exponential model was selected for Haverstraw Bay. The range over which spatial variation was apparent in the data sets varied between 0.62 and 3.87 km or over 16–26% of the maximum extent in the study areas. There was no relationship between scaled values of the nugget and range (product-moment correlation, r = 0.23, p = 0.66).

**Table 6:** Fitted variogram models, total variance estimates (*s*^*2*^), variogram parameter estimates (*c*_0,_ *c*_1_, and *a*), and other variogram relationships for each study location. *c*_0_ is an estimate of the nugget effect and *c*_0_/*s*^2^ is an estimate of the fraction of the total variance represented by small-scale variability. The sum *c*_0_ + *c*_1_ is the sill, and *a* defines the rate and, in the case of the spherical and piecewise linear models, the range at which the sill is reached. For the exponential model, the range was estimated as the distance where the variogram model reached 95% of the sill. The maximum extent is the largest geographic distance between two sampling locations in a study.

Study Location	Fitted Model	*s*^*2*^	*c*_*0*_	*c*_*1*_	*a* (km)	Sill (*c*_*0*_ + *c*_*1*_)	Range (km)	Maximum Extent (km)	Scaled Nugget (*c*_*0*_*/s*^*2*^)	Scaled Range (Range/ Maximum Extent)
Kingston-Saugerties '01	Linear	0.30	0.16	0.16	3.87	0.32	3.87	18.01	0.54	0.22
Kingston-Saugerties '02	Spherical	0.44	0.25	0.22	4.39	0.46	4.39	18.01	0.57	0.24
Haverstraw	Exponential	0.51	0.21	0.32	0.89	0.53	2.22	9.64	0.42	0.23
Tappan Zee	Spherical	0.43	0.18	0.27	0.57	0.45	0.57	4.02	0.42	0.14
Robins	Linear	0.44	0.16	0.32	0.79	0.48	0.79	3.00	0.36	0.26
Shelter	Spherical	0.51	0.19	0.32	0.62	0.51	0.62	3.89	0.37	0.16

### Nonspatial RDA results

**Fig 4:**
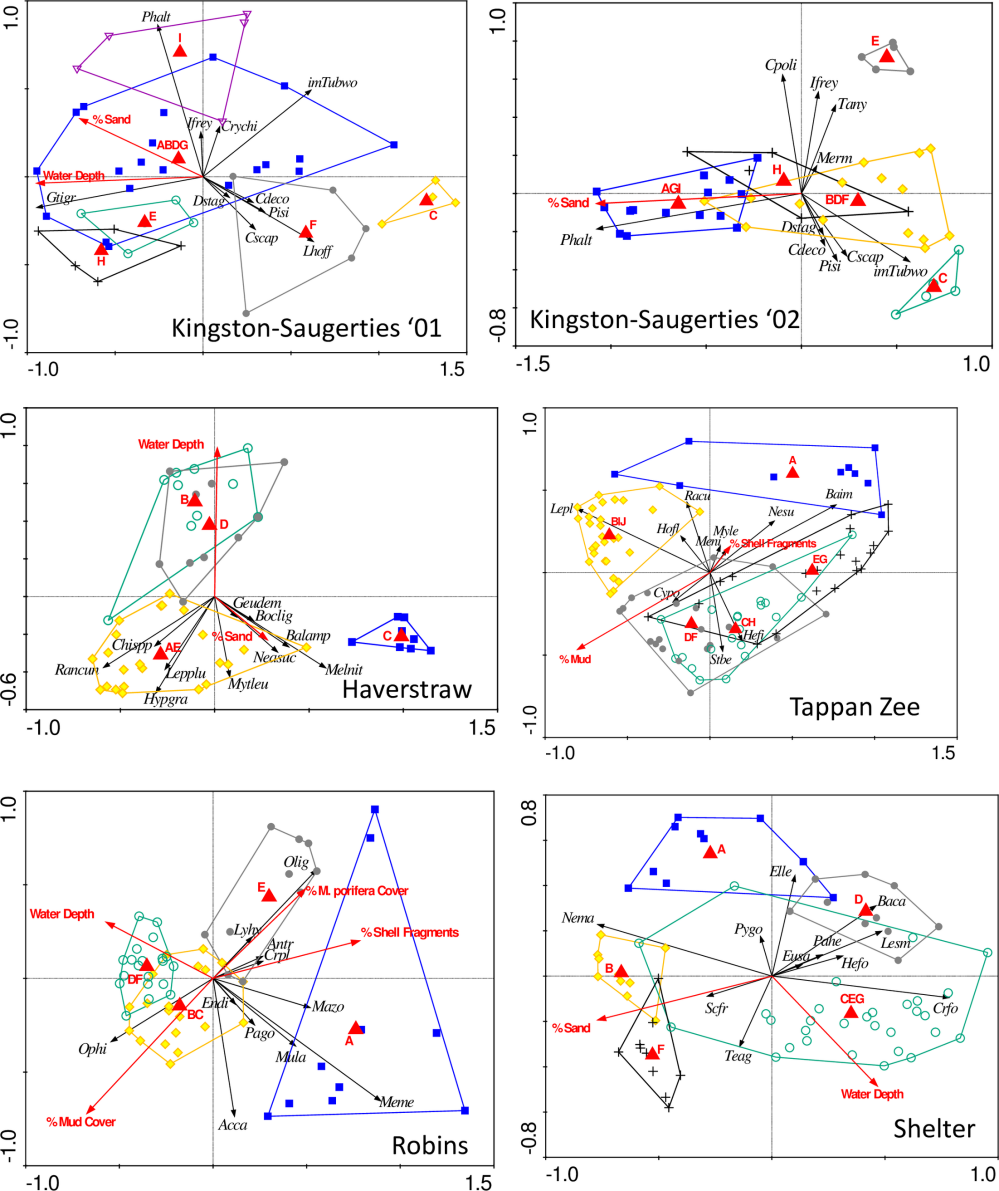
Ordination triplots illustrating the nonspatial RDA results. Scores for individual samples plotted as points. Sample points close to one another tend to have similar faunal structure than those further apart. Different point shapes and color represent samples collected from different acoustic provinces. Polygons enclose samples from each province. Continuous explanatory variables and individual taxa are plotted as vectors. The vector arrowheads represent high, the origin averages, and the tail (when extended through the origin) low values of the selected environmental variables. Projections of sample points onto an individual taxa vector approximate the Hellinger transformed abundances for that taxon. For clarity, only species with the highest amounts of explained variance (typically those with > 10%) are plotted. Acca = *Acteocina canaliculata*, Antr = *Anadara transversa*, Baca = *Batea catharinensis*, Baim = *Balanus imporvisus*, Balamp = *Balanus amphitrite*, Boclig = *Boccardia ligerica*, Cdeco = *Chironomus decorus*, Chispp = Chironomidae sp., Crfo = *Crepidula fornicata*, Crpl = *Crepidula plana*, Crychi = *Cryptochironomus* sp., Cscap = *Coelotanypus scapularis*, Cpoli/Cypo = *Cyathura polita*, Dstag = *Dorylaimus* cf. *stagnalis*, Elle = *Elasmopus levis*, Endi = *Ensis directus*, Eusa = *Eumida sanguinea*, Geudem = *Geukensia demissa*, Gtigr = *Gammarus tigrinus*, Hefi = *Heteromastus filiformis*, Hofl = *Heteromysis formosa*, Hypgra = *Hypaniola grayi*, Ifrey = *Isochaetides freyi*, imTubwo = immature Tubificidae without hair setae, Lepplu/Lepl = *Leptocheirus plumulosus*, Lesm = *Lembos smithi*, Lhoff = *Limnodrilus hoffmeisteri*, Lyhy = *Lyonsia hyalina*, Mazo = *Macroclymene zonalis*, Melnit/Meni = *Melita nitida*, Meme = *Mercenaria mercenaria*, Mula = *Mulinia lateralis*, Mytleu/Myle = *Mytilopsis leucophaeata*, Neasuc/Nesu = *Neanthes succinea*, Nema = Nematode, Olig = Oligochaeta, Ophi = Ophiuroidea, Pago = *Pandora gouldiana*, Pahe = *Panopeus herbstii*, Phalt = *Polypedilum halterale*, Pygo = *Polygordius* sp., Racu/Racun = *Rangia cuneata*, Scfr = *Scoloplos fragilis*, Stbe = *Streblospio benedicti*, Teag = *Tellina agilis*, and Thsp = *Tharyx* sp.

The forward selection process in RDA resulted in the selection of 5 to 7 explanatory variables and r^2^ values representing 42 to 52% of the total variance (Fig 4; Table 7). Acoustic provinces dominated the analysis at all sites, but other environmental variables identified in forward selection and subsequently retained by the AICc model selection criterion were water depth (Kingston-Saugerties’01, Haverstraw Bay, Robins Island, and Shelter Island), percent sand (Kingston-Saugerties’01 and ‘02, Haverstraw Bay, and Shelter Island), percent mud (Tappan Zee) and the percent cover classes from image analysis including % shell fragment cover at Tappan Zee, and % mud cover, % shell fragment cover, and % *M*. *porifera* cover at Robins Island. LOI and RPD were not selected in the RDA.

**Table 7:** RDA forward selection results, eigenvalues, and AICc for each study location. Minimum AICc values are indicated in bold. Capital letters listed in the “Variable” column are provinces, and the variables are listed in order of selection.

Study Location	Variable	Eigenvalue	Sum(Eigenvalue)	AICc
Kingston-Saugerties'01	Water Depth	0.200	0.200	-102.13
	E	0.095	0.295	-105.27
	G	0.084	0.379	-108.30
	H	0.065	0.444	-110.47
	I	0.044	0.488	**-111.26**
	C	0.033	0.521	-111.19
	F	0.033	0.554	-111.15
	B	0.018	0.572	-109.59
Kingston-Saugerties'02	AGI	0.221	0.221	-51.75
	H	0.081	0.302	-54.16
	E	0.075	0.377	-56.60
	% Sand (grain size)	0.061	0.438	-58.45
	C	0.036	0.474	**-58.52**
	LOI	0.021	0.495	-57.31
Haverstraw	C	0.192	0.192	-59.11
	Water Depth	0.115	0.307	-64.58
	% Sand (grain size)	0.038	0.345	-64.99
	B	0.039	0.384	-65.54
	D	0.037	0.421	**-66.01**
	A (or E)	0.019	0.440	-64.89
Tappan Zee	BIJ	0.185	0.185	-183.83
	% Mud (grain size)	0.114	0.299	-196.72
	DF	0.051	0.350	-202.06
	A	0.040	0.390	-206.15
	CH (&EG)	0.017	0.407	-206.66
	% Cover Shell Frag	0.015	0.422	**-206.85**
	Water Depth	0.012	0.434	-206.54
	% Silt-Covered Material	0.004	0.438	-204.77
Robins Island	A	0.148	0.148	-139.13
	% Cover Mud	0.106	0.254	-144.81
	BC	0.080	0.334	-149.23
	% Cover M. prolifera	0.067	0.401	-153.12
	Water Depth	0.043	0.444	-155.02
	E (& DF)	0.041	0.485	-156.94
	% Cover Shell Frag	0.031	0.516	**-157.89**
	% Cover Seaweed (or Crepidula)	0.012	0.528	-156.51
Shelter Island	% Sand (grain size)	0.129	0.129	-172.20
	CEG	0.098	0.258	-178.30
	D	0.060	0.318	-181.63
	A	0.062	0.380	-185.61
	B (or F)	0.046	0.426	-188.26
	Water Depth	0.024	0.450	**-188.54**
	% Cover Rock	0.018	0.468	-188.10

### Multiscale ordination

**Fig 5:**
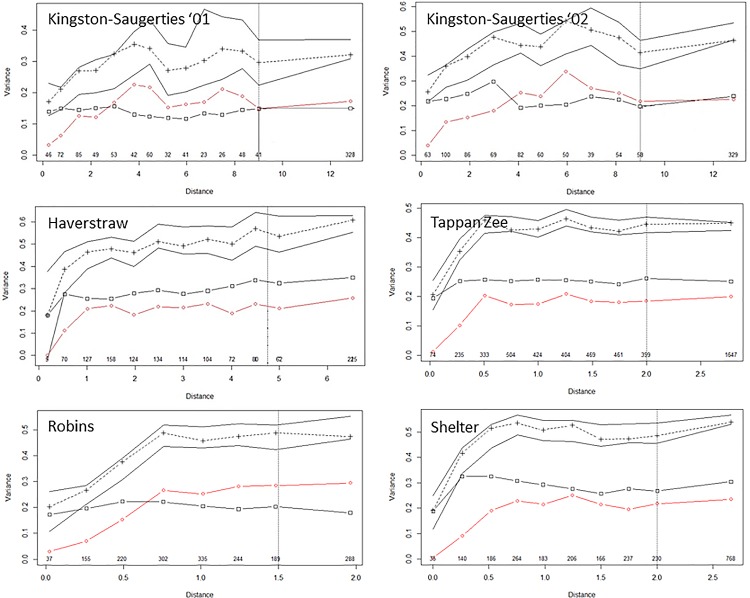
MSO plots for each study area. Crosshairs are the sum of fitted and residual variograms, solid lines are Bonferroni-corrected point confidence envelope of the empirical variogram, open diamonds represent the fitted variogram, and open squares are the residual variogram. Numbers above the distance axis are the number of sample pairs in each distance class. See the explanation of Eq 7 definitions of these quantities. Solid squares, although absent in all cases, would indicate the presence significant spatial autocorrelation in the residuals (detected via Mantel tests). Distances are in kilometers.

MSO results indicated that the spatial dependence in the faunal data was captured by the explanatory variables selected in the RDA, and that the residuals contained no detectible spatial structure (Fig 5). Variograms formed from RDA predictions *γ*_*fit*_(*h*) had evident spatial structure at all sites and strongly paralleled the shape of the empirical variograms, suggesting that the functional form of the RDA model was not misspecified. Points in the variograms formed by the sum *γ*_*fit*_(*h*)+*γ*_*res*_(*h*) lay within the Bonferroni-corrected point confidence interval around *γ*(*h*), indicating that there were no problems with scale dependence in the biotic-environmental relationships. The variogram of *γ*_*res*_(*h*) quickly reached a sill and did not continue to increase with distance class, indicating that the stationarity assumption was met and no unknown environmental factor(s) was present influencing the spatial structure of the residuals. In addition, Bonferroni-adjusted Mantel tests between residuals and a geographic distance matrix at each distance class interval were non-significant, excluding the possibility of autocorrelated residuals.

### RDA performance at local vs. habitat-level scales

**Fig 6:**
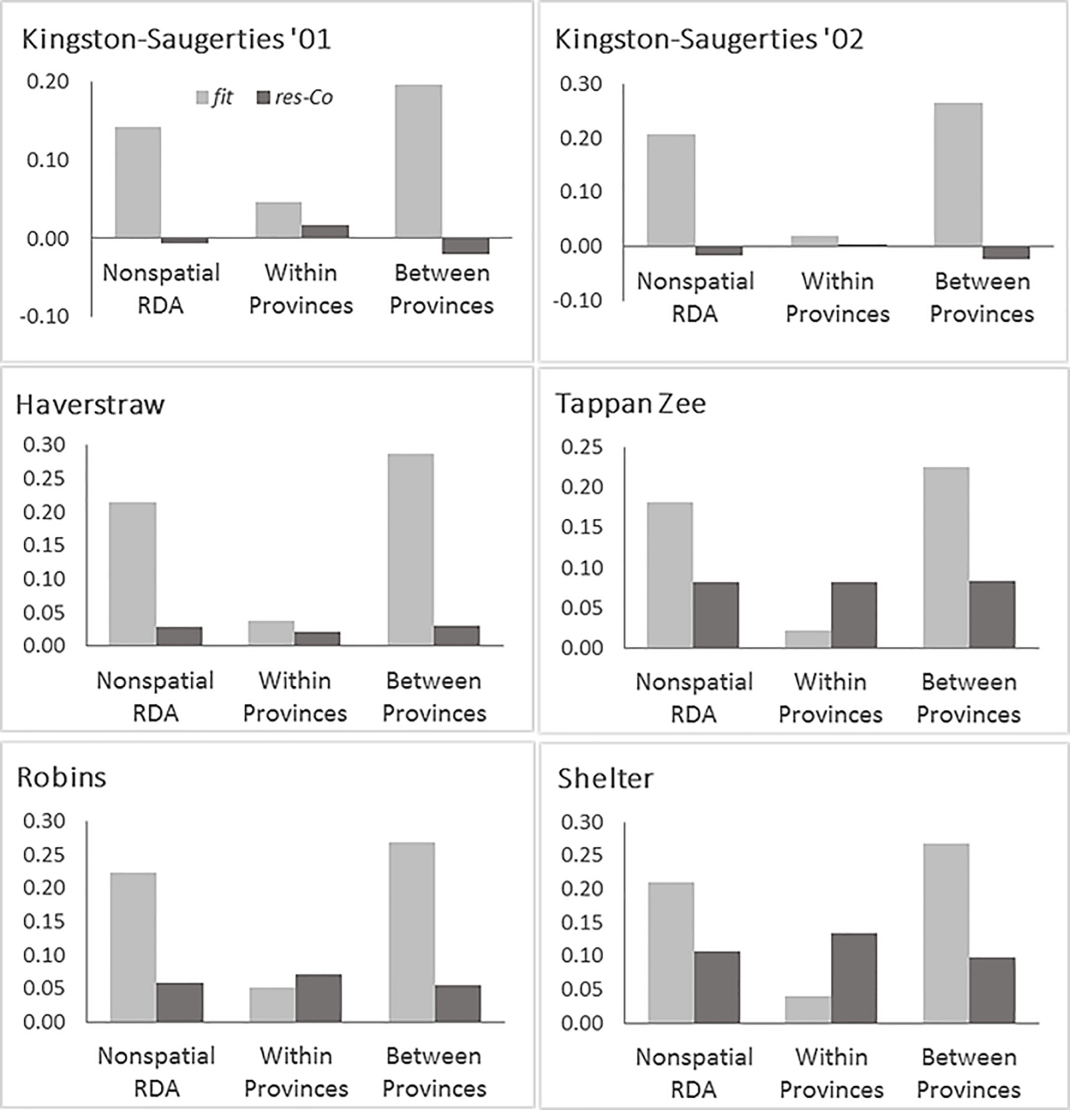
Community variance explained by RDA models (*fit*) compared to nugget corrected residual variance (*res* − *c*_0_). For each data set, comparisons are made for the entire nonspatial RDA and for results broken down into components representing variation within and between acoustic provinces.

Subtracting the nugget estimates of small-scale variability from RDA regression residuals altered the perception of RDA model performance both overall and at local vs. habitat levels (Fig 6). With small-scale variability removed from the residuals, the nonspatial RDA explained >71% of the remaining variance in community structure. When comparisons were restricted to sample pairs within the same acoustic province, the RDA model explained 21–100% of the remaining variance in community structure. The within-province comparisons were notably weak for Haverstraw (33%), Tappan Zee (24%), Robins Island (42%), and Shelter Island (23%). When comparisons were restricted to sample pairs in different acoustic provinces, the RDA model explained >73% of the remaining variance in community structure. Negative residual values in the two Kingston-Saugerties data sets are addressed in the discussion.

## Discussion

In this study, the nugget estimates of small-scale variability represented a substantial proportion of the total community variation across all data sets analyzed (36–59%). The estimates were only about 10% less than the variation present at the smallest distance class in the empirical variograms; thus, even if these observed values were substituted for the nugget effect estimates, the results would not change substantially. Since the nugget estimates were based on the analysis of empirical variograms, they are independent of the multivariate RDA regressions. Potential causal factors for the small-scale variation include biotic interactions [7] and patchiness in settlement [57], especially at a scale approaching the organisms themselves [19], small-scale variability in measured environmental variables, other unmeasured environmental factors that become important at small scales [58,59] and measurement error.

Small-scale variability of this magnitude has been reported in other benthic faunal studies [60–64]. Chapman et al. (2010) [63] examined intertidal macrofauna and microalgae and found that samples 50 cm apart were highly variable and no larger amount of variation was added at scales between 2 and 400 m. They concluded that large-scale processes were less important than small-scale environmental variation in structuring benthic biota. Fraschetti et al. (2005) [60] sampled the rocky intertidal fauna along the Apulian coast of Italy and Greece and concluded that the largest variation was at meter spatial scales relative to 10s to 100s of kilometers. Johnson et al. (2007) [61] determined that > 90% of the variability in salt marsh annelids occurred at < 50m. In their study of soft sediment macrofauna in the Baltic Sea, Kraufvelin et al. (2011) [64] observed most variability at the smallest sampled level (10 m). Sampling infauna off coastal New Jersey at multiple spatial scales, Ramey et al. (2009) [62] found more variability between troughs and crests of sand ripples < 1 m apart than similar features 2 m to 4 km apart. In the current study, large-scale habitat differences in the faunal assemblages were evident. It is striking, therefore, that small-scale variation is still such a dominant characteristic.

A common feature of these benthic studies is the use of a hierarchical nested sampling design and analysis of resulting data by nested ANOVA. This has been a dominant experimental design and analysis approach in marine studies of spatial patterning (see citations in Fraschetti et al. 2005 [60]), especially in the intertidal. It is a powerful technique, one that has led to significant understanding of the relative importance of biotic and physical processes at different scales (e.g., [1,64–67]. It requires *a priori* knowledge of the important spatial scales of variability [68–70] a feature not known for the sites in the current study. It has been shown, for example, that regions derived from backscatter data do not necessarily correspond to distinct faunal assemblages [24,25,71].

It should also be noted that other methods have been developed and successfully applied to examine spatial patterning. Two in particular, spectral analysis [70,72] and Moran’s eigenvector maps [14,52,73] are particularly appropriate for detecting spatial patterns, characterizing them, identifying the characteristic scales if they exist, and relating biotic-abiotic variables by linking them through a spatial structure analysis. The current study had a more limited scope of determining whether a set of commonly collected set of environmental variables accounted for the spatial patterns in the fauna assemblages. Multiscale ordination was well suited to addressing this question.

The combination of commonly used *in situ* and sonar-derived province variables in the RDA regressions consistently accounted for the spatial structure in all of the data sets. The evidence supporting this result is very strong in the variogram analysis where the fitted variogram paralleled the empirical variogram, the residuals at large spatial scales exhibited no trend, and there was no suggestion that the biotic-environmental relationship changed with spatial scale. This outcome held across all six infaunal data sets from markedly different environments ranging from freshwater to near marine conditions. Of the explanatory variables considered, categorical acoustic province variables were dominant in the analyses and accounted for 51–80% of the fitted community variance. This suggests that acoustic provinces were good proxies for habitat in all six of the data sets, a result not wholly expected given the evidence of weak biotic associations with acoustic features found in other studies [23–26]. Strayer et al. (2006) [37] has suggested that large, seascape-scale predictors may function to integrate the dominant controlling processes. The importance of these categorical variables at the six locations examined in this study suggests that benthic infaunal community structure is patchy on a seascape scale, in contrast to a more continuous gradational structure. Additionally, since the scaled range estimated from the data was only 14–26% of the maximum extent of the study areas (Table 6), the surveys were well suited to detect community structure differences driven by large-scale environmental changes.

Assuming that the substantial small-scale variability, as measured by the nugget estimates obtained from the fitted empirical variograms, represents faunal variability below the resolution of the sampling surveys, subtracting it from the residual variance provides a more honest way to assess RDA performance. After doing so, RDA accounted for greater than 71% of the faunal variance remaining (Fig 6). The variance explained was much higher for Robins Island (79%), and probably even greater for the two Kingston-Saugerties data sets. There, estimated small-scale variation exceeded the residual from RDA, resulting in a negative variance. As with any regression analysis, estimates from fitting an empirical variogram and a nonspatial RDA both have uncertainty. Errors in the RDA residual can be assessed by a cross-validation method [74], but unfortunately errors in parameter estimates in fitting empirical variograms have not been evaluated [47]. Perhaps not coincidently, both Kingston-Saugerties data sets had the largest relative nugget estimates, both exceeding 50% of the total faunal variation, and largest absolute standard errors of *γ*(*h*) as evidenced by the confidence intervals in Fig 5. These two data sets also had the least regular transition from within to between province sample pair comparisons with distance (Fig 3). Both the precision and shape of an empirical variogram is known to vary with the configuration of sampling locations [68,69,75], and these factors could certainly have played a role in the nugget effect estimates [76]. In any event, estimates of explained variance at all sites are substantially above an amount that would be considered acceptable in a multivariate study of biotic-environmental relationships.

Extending this small-scale nugget adjustment to comparisons of sample pairs laying within and between provinces (Fig 6) provides some useful insights on the effectiveness of RDA results at local and seascape scales. In comparing pairs of samples from different provinces, RDA accounted for >73% of the remaining faunal variation after nugget adjustment. This again verifies the effectiveness of acoustic provinces as relevant seascape scale explanatory variables. The outcome was more mixed for pairs of samples occurring within the same province. After subtracting the nugget effect, the RDA explained high fractions of the remaining faunal variance (74–84%) for the two Kingston-Saugerties data sets, but it explained relatively low faunal variance at the Haverstraw (33%), Tappan Zee (24%), Robins Island (42%), and Shelter Island (23%) sites.

One potential explanation for the weak within-province results at Haverstraw, Tappan Zee, Robins Island, and Shelter Island is that the fauna were responding to a patchy rather than gradational environmental driver that would have been detected in the residual variogram. If that were the case, one possible avenue of analysis would be to take advantage of the more complete coverage that sonar provides to generate potentially useful explanatory variables. Neighborhood statistics for high-resolution bathymetry and backscatter data are readily available in GIS and specialized sonar processing software such as Fledermaus (Quality Positioning Services, Portsmouth, NH, USA). These applications can estimate a variety of derived, integrated variables such as grey-level co-occurrence measures [77], angular range analysis measures [78], rugosity [79], exposure to wave action and subtidal currents (e.g., aspect and bathymetric position index (BPI)), and vulnerability to sedimentation (slope, BPI). Adjusting the radius surrounding sampling locations when calculating neighborhood statistics is one way to alter the scale of the derived measures. Given the study results to date, this exploration clearly should be focused on assessing within-province rather than between-province variation in community structure.

Accepting the evidence that the study locations are patchy at a seascape-scale defined by the acoustic provinces, the empirical variogram for each site (Fig 3) should be regarded as a complex composite formed from multiple within and between habitat spatial relationships. It is possible that community assemblages within each province have unique and perhaps quite different spatial structures leading to differences in variogram shape, nugget, sill, and range characteristics. In the current study, four of the six variograms appear well behaved with a simple, smooth shape trending to a sill, suggesting that the spatial patterns of the fauna across provinces are similar. The variograms from the Kingston-Saugerties data sets are the exceptions, where the spatial relationships forming the composite variogram seem more complex. The two variograms are similar in shape with pronounced maxima. Sampling locations were repeated in the two seasonal studies at this site, allowing the possibility that the pattern includes an interplay between the actual sample and/or habitat configuration. While some methods exist for estimating variances when combining heterogeneous strata with differing spatial characteristics for univariate data [80] and for combining multiple independent spatial processes within a single region for multivariate data [76], no general method exists for predicting the effects on variance of complex combinations of study area shapes, extents, and sampling intervals [4].

Decomposing a large-scale variogram containing multiple habitats could be approached by a sampling study with a large enough sampling effort to generate empirical variograms for each bottom type. The variogram criteria used in present study required a minimum of 10 distance classes with 30 pairs of samples within each class [47]. Ten distance classes were selected to ensure variance estimates at enough spatial scales to fit a variogram model but not too many to make the Bonferroni-adjusted tests examining RDA results too conservative. Meeting these criteria would require a minimum of 35 sampling locations within each acoustic province to examine within habitat spatial structure. This level of sampling would be substantially greater than the 5–10 samples per province in the present study, and it may be difficult to achieve in practical terms. Sampling density at the sites in the current study ranged from 1.7 to 18 samples per km^2^ and collection and processing effort for each survey required 9 to 18 months. The high cost of sampling is one reason that *in situ* sampling tends to be sparse in many studies [17,22]. Consistent with ecological interpretations of hierarchy theory [6–8,27,28] biotic interactions would tend to become increasingly dominant at smaller spatial scales, so a study at substantially increased sampling density would have to shift emphasis at least in part to assessing biotic processes in addition to measuring small-scale environmental variation. This might begin, therefore, to provide an insight into why the nugget was such a large fraction of the total variance.

None of the six surveys, consisting of 45–100 benthic samples collected across 4–7 habitats in moderate sized areas (3.3 to 16.5 km^2^), suffered from the problem of scale arbitrariness [1]. Spatial structure in the infaunal community was effectively explained by commonly collected environmental variables, and therefore, biotic-environmental inferences were not compromised by the presence of spatial structure in the data. On the other hand, a large fraction of community variation (36–59%) was below the resolution of the surveys and within-province variation was only moderately explained in four of the six data sets. Both these results highlight further challenges to understanding heterogeneity in benthic communities.
